# Shock Resuscitation - the Necessity and Priority of Renal Blood Perfusion Assessment

**DOI:** 10.14336/AD.2022.0105

**Published:** 2022-07-11

**Authors:** Lixia Liu, Yangong Chao, Xiaoting Wang, Chinese Critical Ultrasound Study Group

**Affiliations:** ^1^Department of Critical Care Medicine, the Fourth Hospital of Hebei Medical University, Shijiazhuang 05001, China.; ^2^Department of Critical Care Medicine, the First Affiliated Hospital of Tsinghua University, Beijing 100016, China.; ^3^Chinese Academy of Medical Sciences, Peking Union Medical College, Department of Critical Care Medicine, Peking Union Medical College Hospital, Beijing 100730, China.

**Keywords:** shock, blood pressure, kidney, organ blood flow, autoregulation, critical-care ultrasonography

## Abstract

Improving organ perfusion is the aim of shock resuscitation; therefore, improving organ blood perfusion is a direct indicator for shock resuscitation. During shock, different organs have different capacities for blood flow autoregulation. The kidney is an important organ with excellent ability to autoregulate the blood flow and with vulnerability to poor organ perfusion, which places kidney perfusion in a position of necessity and priority relative to that of other organs in shock. Critical-care ultrasonography provides the best evaluation of renal perfusion.

Shock is an acute circulatory failure, the essence of which is failure to provide tissues with adequate oxygen, leading to cellular hypoxia and organ dysfunction. Shock resuscitation increases oxygen delivery to the tissues and organs by improving tissue perfusion. Oxygen is carried through blood flow, and direct monitoring of changes in the organ’s blood flow is vital in shock resuscitation. Improving organ blood flow is a direct objective in shock resuscitation [1]. However, different organs have unique perfusion pressures and regulatory systems [2] matching their own physiological needs; hence, it is difficult to assess the perfusion status of one organ based on that of a different organ [3]. Therefore, a standardised procedure should be established for grading organ perfusion during shock to guide clinical treatment. The kidney is an important organ with a more comprehensive autoregulation mechanism; however, when compared with the perfusion of the heart, brain, and spinal cord, its perfusion is more susceptible to blood pressure fluctuations. Thus, the evaluation of renal blood perfusion is necessary and should be prioritised over that of the other organs.

## 1. Renal blood flow perfusion is the foundation of renal function

Although the kidney accounts for only 0.4% of the body weight, it receives 20%-25% of the cardiac output (CO). The rationale behind this is not to maintain kidney viability but to filter systemic metabolites from the entire circulation, to regulate water, electrolyte, and acid-base balance, and to maintain internal homeostasis while performing other important functions such as blood pressure regulation. Clearly, normal blood perfusion is the key to ensuring renal function. Decreased renal perfusion reduces the amount of purified blood, the capacity to remove metabolites, and autoregulation by the kidneys. This may manifest as disorders in water, electrolyte, and acid-base balance. Once the damage reaches a certain level, it is clinically defined as an acute kidney injury (AKI).

## 2. Renal vulnerability to decreased perfusion

### 2.1. Renal autoregulation of blood flow is weaker than that in other important organs

Organ perfusion is maintained by CO distribution as a function of organ vascular resistance and arterial blood pressure. The main factors that determine the blood flow perfusion of an organ are three-fold: (i) the level of macrocirculation, *i.e.*, CO; (ii) the organ perfusion pressure, or the gap between the macrocirculatory pressure and the interstitial pressure; this can be replaced with the mean arterial pressure (MAP) when the organ’s interstitial pressure is unknown; and (iii) organ vascular resistance. When CO and MAP are constant, organ vascular resistance determines the organ perfusion and is mainly affected by the organ’s capacity for autonomic regulation of blood flow and by neuroendocrine factors. Except for the pulmonary circulation, which receives all CO without the need for autonomic regulation of blood flow [4], all organs have a certain capacity for autonomic regulation of blood flow [5].

Autonomic regulation of blood flow refers to the maintenance of stable organ perfusion through the organ’s intrinsic capacity to self-regulate vascular resistance as blood pressure or perfusion pressure fluctuates. This reflects the connection between organ perfusion pressure and blood flow. Thus, the greater the regulatory capacity, the higher the probability of stable organ blood flow when blood pressure fluctuates. The stage at which the blood flow does not fluctuate with pressure changes is the *plateau period*, in which the two ends of the plateau period are the lower limit of autoregulation (LLA) and the upper limit of autoregulation (ULA) (5). If the blood pressure or perfusion pressure is above the LLA, not only can the organ be effectively perfused but also the application of vasoactive drugs and fluids can be reduced. An organ with a strong capacity for regulation has a wide and flat plateau stage with a clear LLA, while one with a weak capacity for regulation has a narrow, steep, or even absent plateau period. Regardless of the organ type, the ULA is not easily determined. According to an organ’s capacity for autonomous blood flow regulation, it is classified into three levels: strong (heart, brain, spinal cord, and kidney; among them, the brain and spinal cord have the most powerful and finely tuned autonomic regulation of blood flow); medium (skeletal muscle); and weak (internal organs) (5). In general, organs with weak regenerative capacity have strong blood flow autoregulation, which also indicates the importance of the autonomous regulatory capacity for organ function. In short, automatic regulation of blood flow, to a certain extent, determines the level of organ perfusion during shock.

Some studies have indicated that conditions with poor prognosis, such as multiple organ dysfunction syndrome and sepsis after cardiac surgery in critically ill patients, are correlated with blood pressure lower than the autonomically regulated LLA value for cerebral blood flow; this may serve as a guidance for blood pressure management in critically ill patients [6-8]. However, this principle has been questioned because the autoregulation of cerebral blood flow is superior to that of the kidneys and other organs. When cerebral perfusion is poor, other organs have already experienced decreased perfusion, as the LLA value for cerebral blood flow is lower than that of other organs. Therefore, the application of the LLA value for cerebral blood flow in blood pressure management may lead to hypoperfusion of other organs in some patients [8,9]. Few clinical studies have reported on the autonomous regulatory capacity of cardiac blood flow. When compared with the autoregulatory capacity for blood flow in the heart, brain, and spinal cord, that for renal blood flow is relatively weak, which can also be used to guide blood pressure management in critically ill patients. Therefore, renal blood flow should be evaluated first.

### 2.2. Renal perfusion is more pressure-dependent

The renal circulation is different from that of other organs, in which there are two sets of capillary networks corresponding to different renal functions. The first set is the *glomerular capillary network*, which filters the entire blood volume and forms the second set of capillary networks. Both ends of the glomerular capillary network are the arterioles, which lead to a far greater forward perfusion pressure of the glomerular capillary network than in most other organs (approximately 60 mmHg vs. 10-15 mmHg). Hence, the kidney is more susceptible to MAP fluctuations, which is also why the LLA is relatively high. In addition, this feature keeps the renal resistance vessels in a state of constant high pressure; therefore, the kidney’s capacity for autonomous regulation of blood flow is easily impaired. In the setting of hypertension and diabetes, damage to the autoregulatory capacity is accelerated, so that the blood flow autoregulation curve shifts to the right and the LLA increases. The results of the SEPSISPAM study showed that compared with an MAP of 65-70 mmHg, that of 80-85 mmHg reduces the serum creatinine levels and renal replacement therapy requirements in patients with septic shock and pre-existing hypertension [10]. Similarly, Poukkanen et al. [11] discovered that an MAP value of <73 mmHg in patients with sepsis is correlated with the occurrence and progression of AKI; almost half the patients in the study had a history of hypertension.

The second set of capillaries are *peritubular*, and to reabsorb most of the glomerular filtrate, the intra-peritubular capillary pressure must be adequately low. In general, the effective glomerular filtration pressure is approximately 10 mmHg; if the intra-peritubular capillary pressure is above this, reabsorption stops. Due to the low pressure of this system, it is easily susceptible to ambient pressures, such as intra-abdominal pressure, renal interstitial pressure, and especially renal venous pressure. The renal venous pressure is the direct resistance of peritubular capillary blood flow back. If the renal venous pressure increases, which is approximately 3-4 mmHg normally, the peritubular capillary pressure increases and reabsorption from the renal tubule decreases. Through backward conduction of pressure, increased renal venous pressure can decrease glomerular filtration pressure, increase interstitial pressure, and decrease renal perfusion. A slight increase in renal venous pressure leads to an increase in renin release, resulting in increased renal vascular resistance and reduced renal perfusion [12]. Under these circumstances, to increase renal perfusion, higher forward perfusion pressure is required, which makes the kidney more susceptible to fluctuations in MAP and leads to the occurrence and progression of AKI [13,14]. The renal venous pressure must be at least 2 mmHg higher than the central venous pressure (CVP) in the supine position for renal venous blood to return to the inferior vena cava [15]; hence, elevated CVP is the most direct factor affecting renal venous pressure and is an independent risk factor for the occurrence and progression of AKI [16].

### 2.3. The kidneys are more susceptible to neuroendocrine factors than are the heart, brain, and spinal cord

Under normal circumstances, the autonomous regulation of organ blood flow is mainly through metabolism. Vasodilators generated by metabolism dilate the blood vessels [17] and increase blood flow, as in the brain during thinking, skeletal muscle during exercise, and digestive organs during eating. In hypotension, although autonomous regulation is essential for blood flow homeostasis, its dominant role is replaced by stimulation of sympathetic nervous system reflexes triggered by hypotension. Hence, blood flow to the organs is redistributed according to the vascular adrenergic receptor density and reactivity in local tissues. Blood vessels of the skin are rich in α-adrenergic receptors, followed by those of the gastrointestinal tract and kidneys. Although α-adrenergic receptors are also present in the cardio-cerebral vessels, they are sparsely distributed; more importantly, they have other internal vascular regulatory mechanisms that dilate blood vessels and increase oxygen uptake [18,19]. Therefore, during shock, blood flow is diverted from the skin, skeletal muscles, and splanchnic organs to the brain, heart, and kidneys [20]. If the shock is severe or persistent, the renal blood vessels constrict and shunt their blood flow to the heart, brain, and spinal cord. Under these circumstances, blood flow to the kidneys and other organs is significantly reduced, even to 1/4 to 1/3 of normal flow, while that to the heart, brain, and spinal cord remains unchanged. Distributive shock, such as septic shock, is somewhat different from other types of shock; due to vascular paralysis, organ perfusion may not decrease in the early stage of shock [21,22]. As mentioned earlier, both ends of the glomerular capillary network are arterioles that are more susceptible to distribution factors, especially the efferent arterioles, which are more dilated than the afferent arterioles, resulting in decreased effective filtration pressure, decreased glomerular filtration, and impaired renal function [23]. Under these circumstances, early titration of vasoconstrictor medications to increase MAP above the LLA may ensure effective renal filtration pressure. Because the LLA of the kidneys is higher than that of the heart, brain, and spinal cord, the MAP value at that time is also suitable for the heart, brain, and spinal cord, provided there are no primary vascular diseases in those organs.

In summary, renal blood perfusion is more dependent on pressures such as MAP and CVP, and its LLA is high. During shock, the kidney is more susceptible to damage than are the other vital organs such as the heart, brain, and spinal cord. This characteristic indicates that changes in renal blood flow can better reflect macrocirculatory changes in the early stages. Determining the LLA is relatively straightforward and can help in the management of pressures and blood flow.

## 3. Renal blood flow is easily evaluated and monitored

The heart and lungs supply oxygen, and cardiopulmonary function and perfusion require special attention. Coronary angiography is a common method for assessing cardiac perfusion. Due to its invasiveness, its utilisation and promotion have been limited and mostly used in patients with coronary artery disease. Pulmonary perfusion is a direct measure of CO; however, as the pulmonary circulation receives all CO and lacks the ability to autoregulate blood flow, it is a poor indicator of perfusion of the other organs [4]. The brain is a critically important organ, and bedside Doppler ultrasonography is a conventional method to evaluate cerebral blood flow; however, the skull limits the routine evaluation of global brain perfusion.

Consider the organs with weak autonomic regulation of blood flow. Circulation to the skin and sublingual mucosa, which have weak autonomic regulation and abundant α-adrenergic receptors, is often sacrificed early in shock. Thus, assessing perfusion of these areas is important; however, due to the weak autonomic regulation of blood flow, it is difficult to extrapolate perfusion changes in these locations to those of other organs. Because the LLA value is unclear, it is rarely used as guidance for pressure management.

Studies on renal perfusion assessment have been relatively mature and have mainly focused on non-invasive methods such as ultrasonography, dynamic contrast-enhanced magnetic resonance imaging (MRI), isotopic renography, and computed tomography (CT) perfusion imaging. Because of the lack of a linear relationship between MRI signal intensity and contrast agent concentration [24], the radiation exposure of isotopic renography and CT perfusion imaging, high costs, and unsuitability for real-time bedside evaluation and monitoring, the application of these methods in critically ill patients is very limited [25,26]. Ultrasonography allows bedside evaluation and monitoring of global renal blood perfusion, including changes in the renal artery, microcirculation, and venous blood flow. The Critical-Care UltraSonography Guided (CCUSG)-‘A_(KI)_BCDE’ protocol proposed by the Chinese Critical UltraSound Study Group [27] adopts two-dimensional renal blood flow distribution, renal arteriovenous blood flow spectrum, renal resistive index (RRI), venous impedance index (VII), and contrast-enhanced ultrasonography (CEUS) to comprehensively evaluate renal perfusion and guide treatment.

First, through renal blood flow distribution, information regarding the amount of blood flow and the perfusion area can be acquired directly. The renal arterial and venous spectral waveforms and corresponding RRI and VII can reflect the perfusion resistance and return resistance of the kidney, respectively, to a certain extent. Several factors influence the correlation between RRI and vascular resistance, such as vascular compliance, distal vascular bed cross-sectional area, and heart rate [28]. Vascular compliance directly determines the influence of resistance on RRI [29]; therefore, it limits the role of RRI in guiding the titration of blood pressure treatment in patients with shock [30]. However, a series of studies have confirmed that RRI is closely related to pulse pressure and MAP [31-33]. Lerolle et al. [34] and Beloncle et al. [35] found that RRI and MAP are inversely proportional in septic shock, especially in patients with hypertension and diabetes, and this phenomenon is remarkable. These studies suggest that the correlation between MAP and RRI can be applied to determine the renal LLA value adjusted by autonomic regulation for renal perfusion pressure titration [34-36], thus improving renal perfusion. The high compliance of the renal vein renders it susceptible to the influence of ambient pressure, especially CVP [16]. In this circumstance, the renal vein spectrum is discontinuous and VII increases even earlier than that of the inferior vena cava [37]. However, the global kidney circulation cannot fully represent the renal microcirculatory perfusion, especially in the stage of septic AKI, and CEUS can dynamically display not only the speed and amount of microcirculatory perfusion but also its clearance in real time and can further guide perfusion pressure titration and other treatments [38-40]. At present, studies on renal haemodynamic changes during shock are ongoing, and no definitive conclusions have been drawn. Moreover, the renal hemodynamic changes very greatly there is great variation among patients, which makes it necessary to “evaluate what is happening before giving treatment”.

## 4. Renal perfusion assessment must be combined with that of other organs

**Figure 1.**
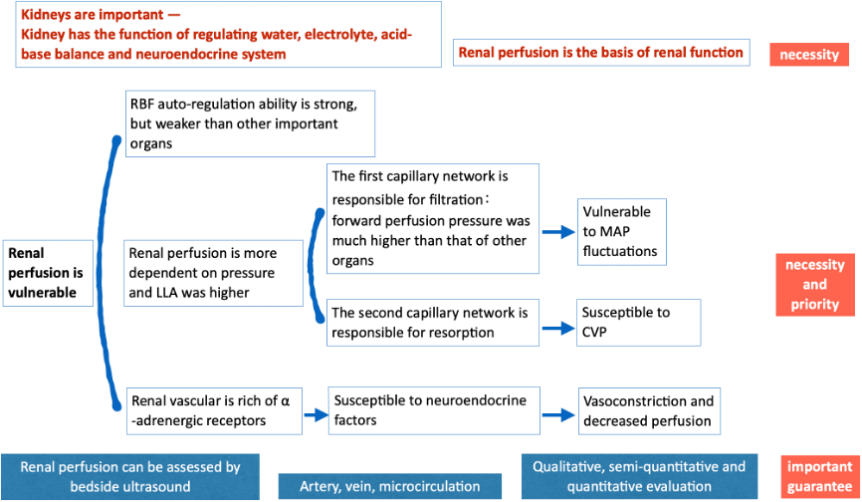
Shock resuscitation- the necessity and priority of renal blood perfusion assessment.

The necessity and priority of renal perfusion assessment proposed in this study do not imply that the kidney is more important than other important organs. The kidney has good ability to autoregulate blood flow; however, it also has the characteristics of the more vulnerable organs with weaker autoregulation. Therefore, during shock, the kidneys can reflect macrocirculatory changes earlier than the other important organs, such as the heart, brain, and spinal cord, and assist in titration of perfusion pressure. If renal perfusion is improved, the perfusion of the heart, brain, and spinal cord should also be improved. When patients have cardio-cerebrovascular disease—that is, when the autonomous regulation of cardio-cerebral blood flow is dysfunctional—the evaluation for cardio-cerebral perfusion should not only be emphasised but also prioritised. When conflicts exist with cardio-cerebral perfusion, perfusion of the heart and brain should be guaranteed and optimised as the first priority (Fig. 1).

In conclusion, optimal autoregulation of renal blood flow and adequate perfusion are important to guarantee that the kidney can regulate water, electrolytes, acid-base balance, and neuroendocrine function. This serves as the essential basis for evaluation of renal perfusion in shock. The kidneys have weaker autonomous regulatory capacity and are more sensitive and dependent on pressures, such as MAP and CVP, than the heart, brain, and spinal cord. Hence, the LLA value tends to be higher, contributing to its vulnerability to damage, and this vulnerability further justifies the necessity and priority of renal evaluation. The LLA of the kidneys, which is relatively clear and higher than that of the heart and brain, is the goal of MAP optimisation after initial shock resuscitation. Bedsides, ultrasonography can comprehensively evaluate renal blood perfusion and guide LLA titration, which is essential in the evaluation of organ perfusion in shock. This blood pressure level is not the final goal of shock resuscitation and must be further titrated to ensure perfusion of the other organs with weaker autonomic blood flow regulatory capacity, such as the gastrointestinal tract and skin. Therefore, further assessment of cardio-cerebral perfusion is needed in cases of cardiovascular and cerebrovascular diseases; however, these still must be based on renal perfusion assessment.
